# Improved extraction efficiency of CsPbBr_3_ perovskite light-emitting diodes due to anodic aluminum oxide nanopore structure

**DOI:** 10.1038/s41598-022-19074-y

**Published:** 2022-08-30

**Authors:** Lung-Chien Chen, Chien-Hong Kao

**Affiliations:** Department of Electro-Optical Engineering, National Taipei University of Technology, Taipei, 10608 Taiwan

**Keywords:** Nanoscience and technology, Optics and photonics

## Abstract

In this work, we investigate the improvement in the performance of a CsPbBr_3_ perovskite light-emitting diode (PeLED) due to an anodic aluminum oxide (AAO) nanopore structure. The AAO structure in the CsPbBr_3_ PeLED structure can improve the light extraction efficiency of CsPbBr_3_ PeLEDs in two ways: the emission light in the side direction being redirected to the normal direction due to the light scattering effect caused by aluminum oxide nanopores and the effective emission area as a result of the rough surface of the AAO structure. The peak luminance, current efficiency, and external quantum efficiency (EQE) were 11,460 cd/m^2^, 2.03 cd/A, and 0.69% at a bias of 6.0 V, respectively. For comparison, the luminance, current efficiency, and EQE values of CsPbBr_3_ PeLEDs with the AAO structure using 50 V of pore-expanding voltage demonstrated improvements of 282%, 190%, and 1280%, respectively, over CsPbBr_3_ PeLEDs without the AAO structure.

## Introduction

Metal halide perovskites can be prepared from solution in an easy and costly method. They exhibit better thermal and chemical stability, improved luminescent color saturation, high photoluminescence quantum yields (PLQYs), narrow full width at half maximum (FWHM), and good spectral tenability, so they have received significant attention. To improve the brightness and stability of perovskite light-emitting diodes (PeLEDs) so that they can achieve practical purposes, a common method is to add additives or ligands to modify the interface or surface in the PeLED structure to reduce surface defects^1–10^.

Anodic aluminum oxide (AAO) templates have been applied in LED structures to improve the light extraction efficiency by increasing the emitting area of LEDs^11–13^. In 2017, Demchyshyn's team used nanoporous silicon and aluminum oxide films as templates. By reducing the pore size, it was found that the photoluminescence (PL) had a significant blue-shift, making the infrared shift to red. In addition to limiting the particle size to adjust the emission wavelength, confinement of perovskite nanocrystals in porous alumina films can significantly improve photoluminescence stability because alumina templates can be used for encapsulation. The results show that the turn-on voltage of the LED is approximately 2.5 V, with a current efficiency of 0.09 cd/A and an external quantum efficiency of 0.03%, while the PLQY of perovskite nanowires can be as high as 90%^14^. In 2020, Lin et al*.* used a combination of inkjet printing and nanoporous anodic aluminum oxide (AAO) for lasers and wide color gamut phosphors to fabricate printed perovskite nanowires (NWs). Due to the presence of AAO templates, the compact space confinement within and the perovskite encapsulation process combined with a highly stable emission intensity. It was only 19% lower after 250 h of continuous excitation with 30 mW/cm^2^ UV and only 30% lower after storage for 3 months in 50% humidity air^15^. In 2017, Waleed's team synthesized nanowires (NWs) in anodized aluminum oxide films by a chemical vapor deposition (CVD) method. The results showed that the small size of anodized aluminum oxide increased the surface area of perovskite, and the high surface energy contributed to stabilizing the cubic phase. Additionally, anodized aluminum oxide protects against the invasion of water and oxygen molecules, which can significantly improve the lifetime of perovskites^16^. Therefore, in this work, we studied the characteristics of CsPbBr_3_ perovskite light-emitting diodes with anodic aluminum oxide nanopore structures.

## Experimental procedure

### Materials and precursors

NiO_x_ powder (0.923 g) was added to 10 mL of ethylene glycol, 0.67 mL of ethylenediamine, and 0.60 mL of ethanolamine and then stirred at 500 rpm overnight to obtain a NiO_x_ solution. PEO powder (0.01 g) was added to 1 mL DMSO and then stirred at 700 rpm at 70 °C for 60 min to obtain a polyethylene oxide (PEO) solution. CsBr powder (0.0958 g) and PbBr_2_ powder (0.1101 g) were added to 1 mL of DMSO solution and then stirred at 750 rpm at 140 °C for 30 min to obtain a CsPbBr_3_ solution. Next, the CsPbBr_3_ solution was mixed with 0.4 mL PEO solution and 1 mg TPBi powder and then stirred at 750 rpm overnight to prepare the CsPbBr_3_:(PEO, TPBi) precursor.

### Device fabrication

The FTO glass substrates were washed using acetone and ethanol for 10 min each and UV ozone treatment for 15 min, sequentially, to remove organic impurities and moisture. The 30-nm-thick NiO_x_ layer as a hole injection layer was spin-coated on FTO glass substrates at 4500 rpm for 90 s and annealed in air at 350 °C for 10 min. The 200-nm-thick Al film was evaporated on FTO/NiO_x_ substrates. Next, the AAO process was carried out by using oxalic acid (0.3 M) as the electrolytic solution, FTO/NiO_x_/Al as the anode, and a carbon rod as the cathode, and then the substrates were put into the H_3_PO_4_ solution for 12 min to expand the pores to form an AAO template with through-pores. Sequentially, the FTO/NiO_x_/AAO template substrate was transferred into a N_2_-filled glove box.

**Figure 1:**
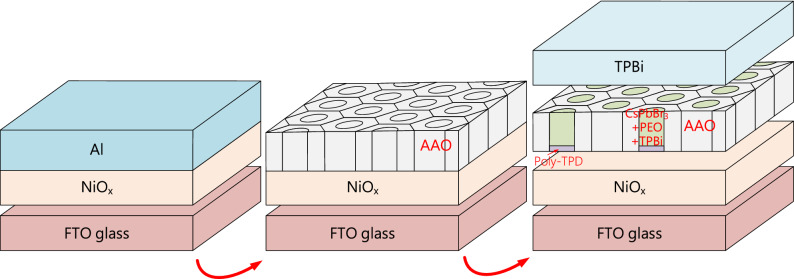
Schematic diagram of the device process flow.

The poly-TPD solution was spin-coated on the FTO/NiO_x_/AAO template substrates at 4000 rpm for 60 s and annealed at 100 °C for 10 min to form the hole transport layer. The perovskite CsPbBr_3_:(PEO, TPBi) blend solution was spin-coated at 3000 rpm for 60 s and annealed at 80 °C for 10 min to form an FTO/NiO_x_/poly-TPD/CsPbBr_3_:(PEO, TPBi) structure with a 150-nm-thick active layer. Finally, a 10-nm-thick TPBi electron transport layer and 100-nm-thick Ag cathode were sequentially deposited by a thermal coater to complete the CsPbBr_3_ PeLED with an AAO structure. The active area of the CsPbBr_3_ PeLEDs with an AAO structure was determined to be 4 mm^2^. Figure 1 illustrates the schematic diagram of the device process flow.

### Characterization

The characteristics of the materials and devices in this work were measured by a PANalytical X'Pert PRO MRD diffractometer (Almelo, The Netherlands) for phase identification, a Hitachi F-7000 fluorescence spectrophotometer (Tokyo, Japan) for the optical properties, a ZEISS Sigma field emission scanning electron microscope (FESEM) (Munich, Germany) for the top view and cross-sectional images, and a Photo Research spectroradiometer PR-670 (Syracuse, NY, USA) for the optoelectronic parameters PeLEDs.

## Results and discussion

**Figure 2:**
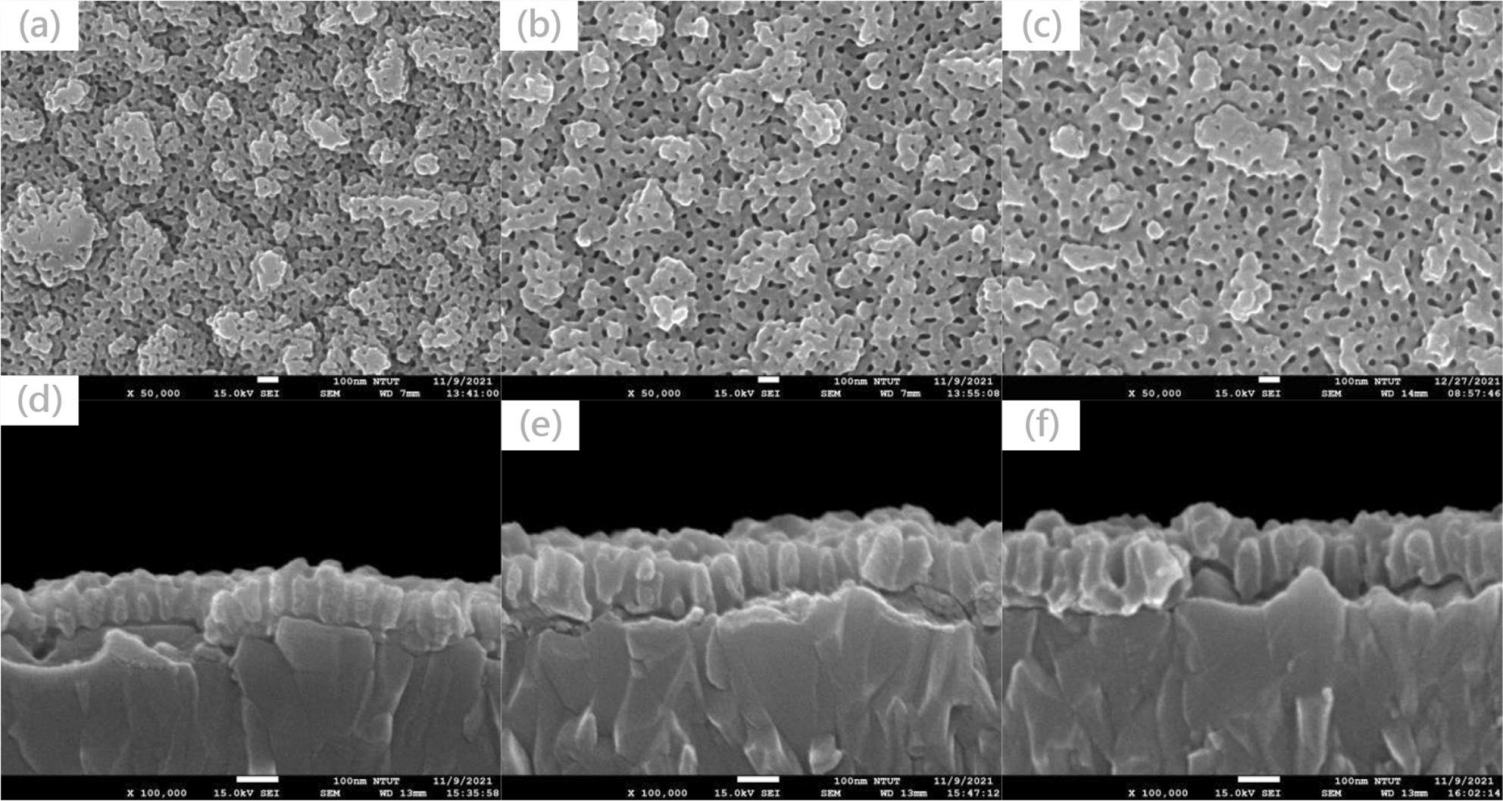
Top view and cross-sectional SEM images of AAO with various pore-expanding voltages: (**a**,**d**): 30 V, (**b**,**e**): 40 V; and (**c**,**f**): 50 V.

To allow the CsPbBr_3_ perovskite solution to flow into the pores of AAO, after the AAO film was completed, the pores were expanded by H_3_PO_4_ for 12 min. Figure 2 shows the top view and cross-sectional SEM images of AAO with various pore-expanding voltages. The average pore sizes of the AAO structures with pore-expanding voltages of 30, 40, and 50 V are 28.57, 32.14, and 37.71 nm, respectively. As the applied voltage increases, the pore size increases and most effectively removes the lower-most AAO layer so that the AAO does not block the CsPbBr_3_ perovskite layer from forming on top of the poly-TPD hole transport layer, thus allowing the injection current to flow through. Also, the thickness increases and to be contributive to extraction more ray from PeLED inside^17^.

**Figure 3:**
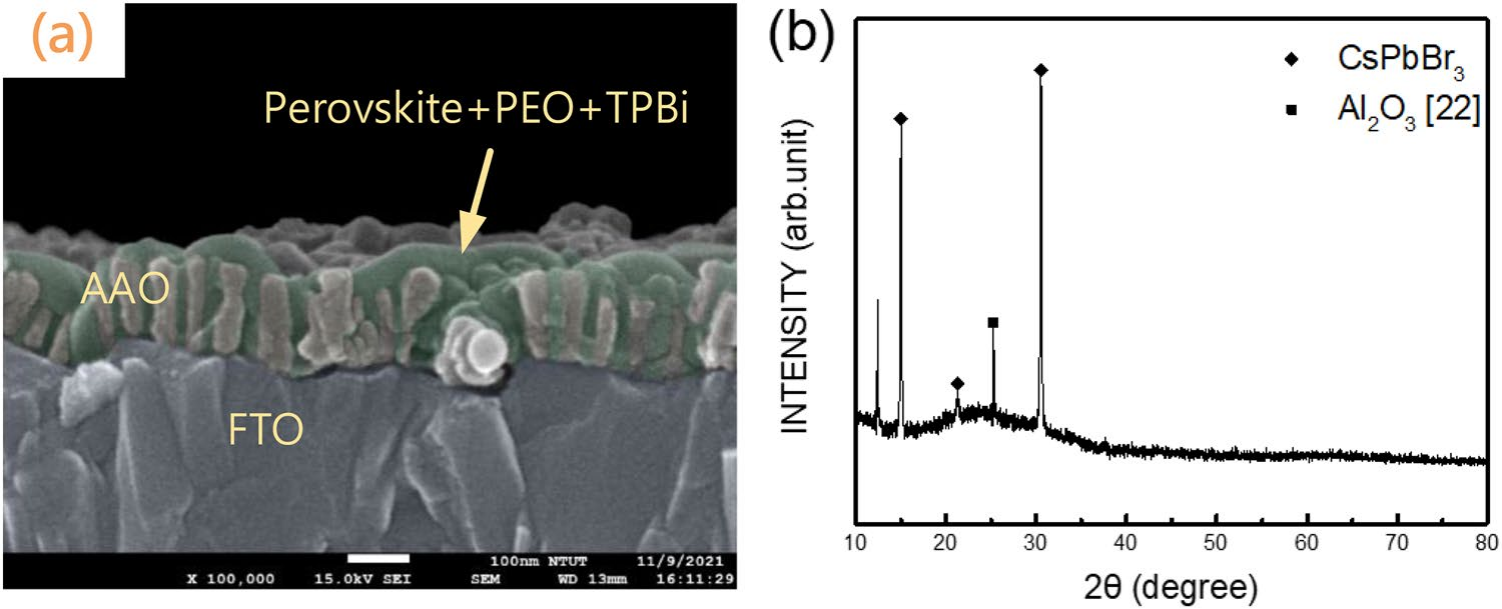
(**a**) Cross-sectional SEM image and (**b**) XRD pattern of the CsPbBr_3_ perovskite film coated onto and into AAO films.

Figure 3a shows the cross-sectional SEM image of the CsPbBr_3_ perovskite/AAO structure. Observably, the CsPbBr_3_ perovskite film covered and penetrated the AAO film. Figure 3b plots the XRD pattern of the CsPbBr_3_ perovskite film coated onto and into the AAO film. In terms of XRD analysis, as shown in Fig. 3b, the XRD characteristic peaks of perovskite CsPbBr_3_ are approximately 15°, 21°, 31.1°, corresponding to the (101), (121), and (202) phases of the cubic lattice structure, respectively^18–20^. Additionally, there is a clear peak at approximately 2θ = 13.1°, which corresponds to the lattice plane of (101), presumably because it is produced by the protruding orthorhombic planes of perovskites^21^.

**Figure 4:**
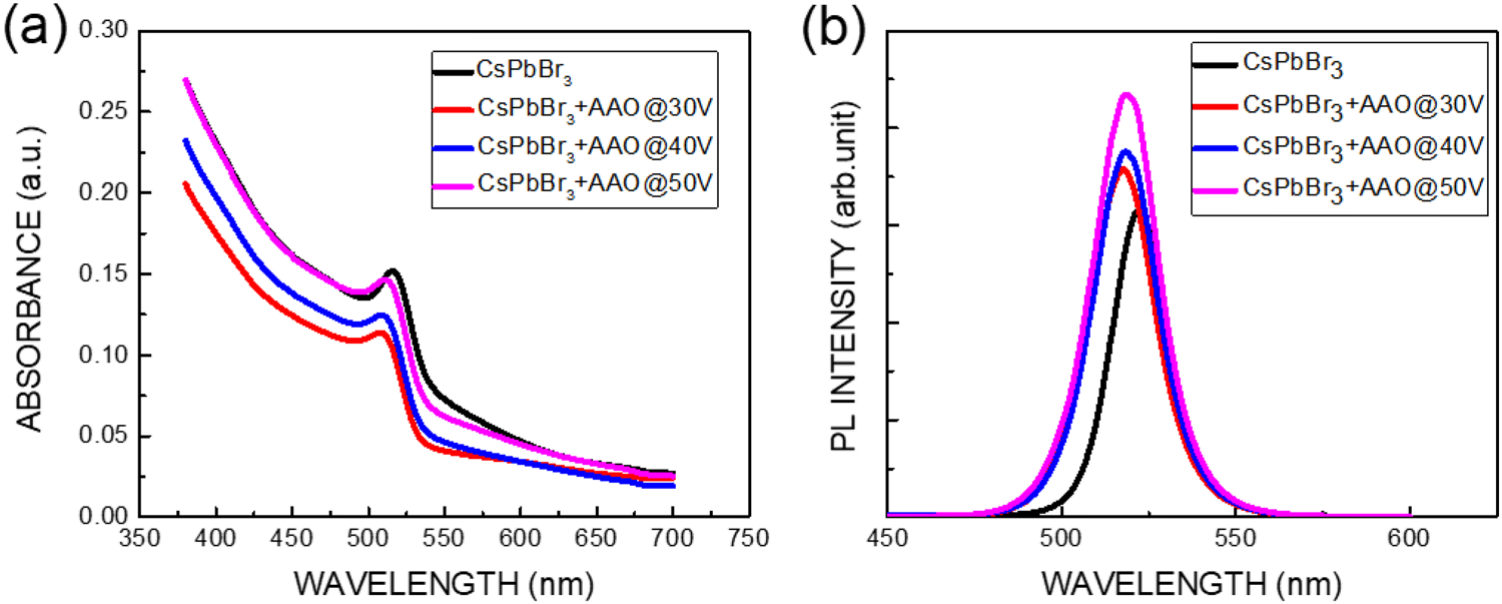
(**a**) Absorbance and (**b**) photoluminescence (PL) spectra of CsPbBr_3_ perovskite film on and in AAO films.

Figure 4 plots the absorbance and photoluminescence (PL) spectra of the CsPbBr_3_ perovskite film on and in AAO films. The absorption edges of the CsPbBr_3_ perovskite film with and without AAO films are 510 nm (2.431 eV) and 514 nm (2.412 eV), respectively, and the peaks of the PL spectrum of the CsPbBr_3_ perovskite film with and without AAO films are 518 nm (2.394 eV) and 522 nm (2.375 eV), respectively. The blue-shift of approximately 8 nm between the absorbance and PL spectra for the CsPbBr_3_ perovskite film with and without AAO films may be due to the Stoke shift^22,23^. The energy shift between absorbance and PL spectra is 37 meV, corresponding to the $\frac{3}{2}kT$, where k is Boltzmann constant, and T is temperature). Therefore, the Stoke shift of 8 nm is caused by the thermal energy form the excitation laser. The blue-shift of approximately 4 nm for the CsPbBr_3_ perovskite film with and without AAO films may be caused by the pore size effect caused by the bandgap modulation^23–25^.

**Figure 5:**
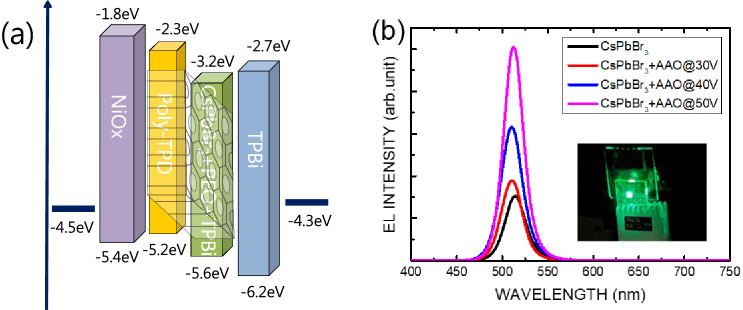
(**a**) Diagram of the electron level of a CsPbBr_3_ PeLED with AAO. (**b**) Electroluminescence (EL) spectra of the CsPbBr_3_ PeLEDs with and without AAO structures for various pore-expanding voltages. The inset of (**b**) is a photo of the CsPbBr_3_ PeLED with an AAO structure and a pore-expanding voltage of 50 V.

Figure 5a sketches the diagram of the electron level of the CsPbBr_3_ PeLED in this work. Figure 5b plots the electroluminescence (EL) spectra of the CsPbBr_3_ PeLEDs with and without the AAO structure for various pore-expanding voltages. The inset of Fig. 5b is a photo of the CsPbBr_3_ PeLED with an AAO structure and a pore-expanding voltage of 50 V operating at a bias of 6 V. The peak position of the EL spectrum of the CsPbBr_3_ PeLED without an AAO structure is 514 nm. The peak position of the EL spectrum of CsPbBr_3_ PeLEDs with an AAO structure with 30 and 40 V of pore-expanding voltages is 510 nm. The peak position of the EL spectrum of the CsPbBr_3_ PeLED with an AAO structure with a 50 V pore-expanding voltage is 512 nm. The blue-shift is consistent with the results of the PL spectra. The blue-shift of approximately 2–4 nm between the CsPbBr_3_ perovskite film with and without AAO structure may be caused by the pore size effect owing to the size issue of the CsPbBr_3_ perovskite nanowires in AAO structure and CsPbBr_3_ perovskite film status^24–26^.

**Figure 6:**
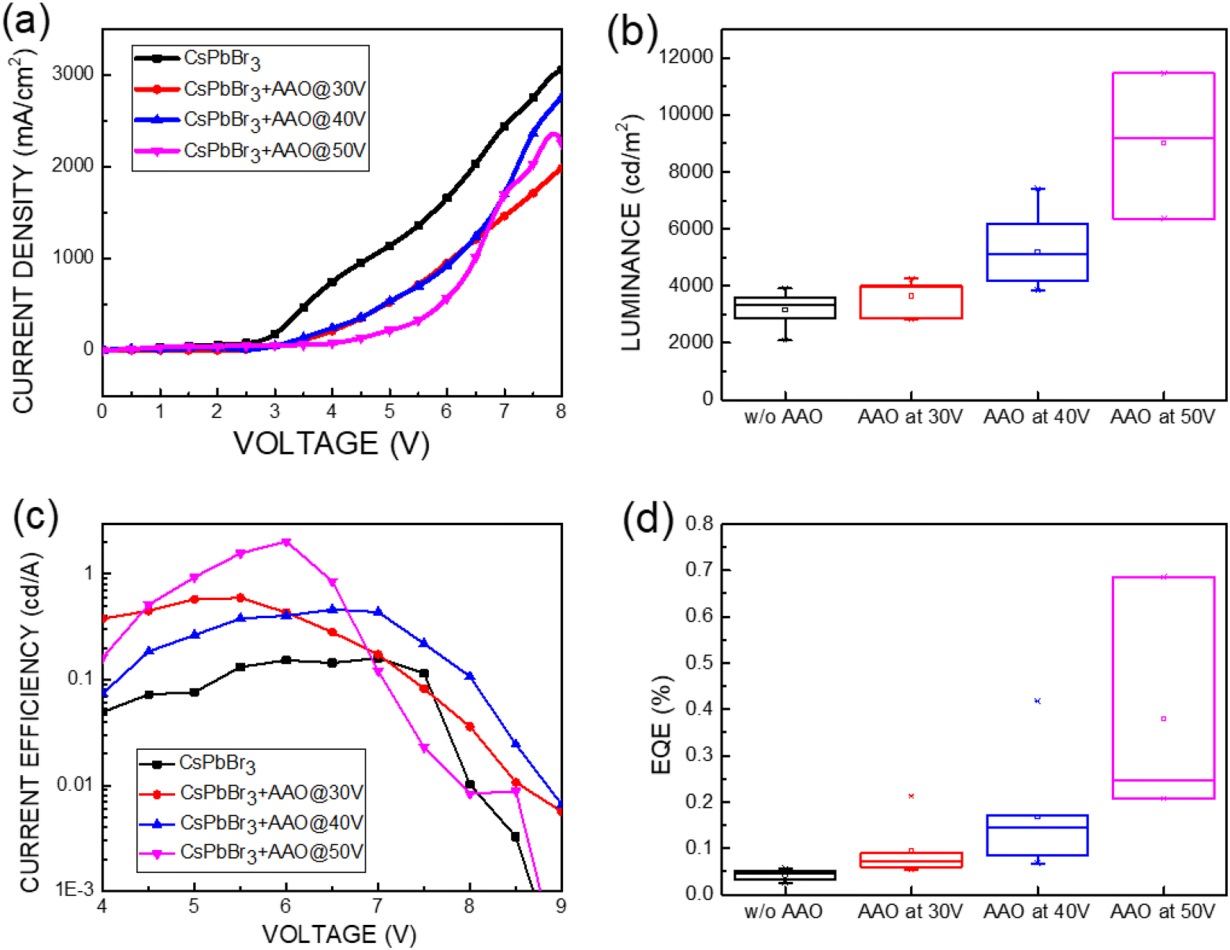
(**a**) Current density, (**b**) luminance, (**c**) current efficiency, and (**d**) external quantum efficiency (EQE) of CsPbBr_3_ perovskite LEDs with and without AAO structures.

**Figure 7:**
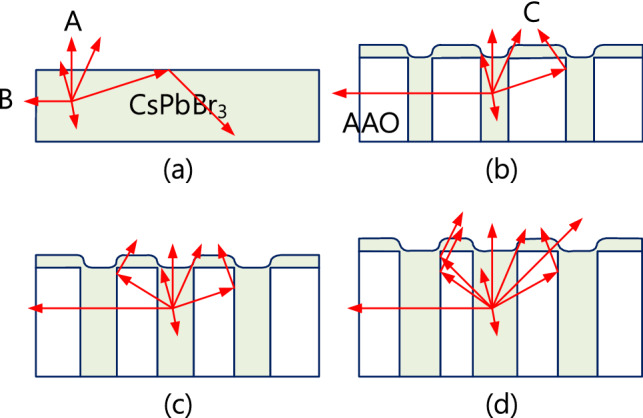
Emission diagram of the CsPbBr_3_ active layer without and with the AAO structure using expanded pore treatment: (**a**) without the AAO structure, (**b**) with the AAO structure using 30 V treatment, (**c**) with the AAO structure using 40 V treatment, and (**d**) with the AAO structure using 50 V treatment.

Figures 6a–d show the current density, luminance, current efficiency, and external quantum efficiency (EQE) of CsPbBr_3_ perovskite LEDs (PeLEDs) with and without an AAO structure. As shown in Fig. 6a, when the AAO structure was applied to the CsPbBr_3_ PeLED structure, the turn-on voltage of the PeLEDs increased from 3 to 4 V due to a reduction in the contact area between the NiO hole injection layer and the poly-TPD hole transport layer. The luminance, current efficiency, and EQE of CsPbBr_3_ PeLEDs without an AAO structure are approximately 3000 cd/m^2^, 0.1 cd/A, and 0.05%, respectively at bias of 6 V. The luminance of CsPbBr_3_ PeLEDs with an AAO structure was superior to that of CsPbBr_3_ PeLEDs without an AAO structure, even if the turn-on voltage increased owing to the reduction in contact area. As shown in Fig. 6b–d, the luminance of CsPbBr_3_ PeLEDs with the AAO structure and pore-expanding voltages of 30, 40, and 50 V were approximately 4000, 6000, and 10,000 cd/m^2^, respectively, at bias of 6 V. The current efficiency of CsPbBr_3_ PeLEDs with an AAO structure and pore-expanding voltages of 30, 40, and 50 V was approximately 0.5, 0.4, and 2 cd/A, respectively. The EQE of CsPbBr_3_ PeLEDs with AAO structures and pore-expanding voltages of 30, 40, and 50 V was approximately 0.1, 0.2, and 0.7%, respectively. The peak luminance, current efficiency, and EQE were 11,460 cd/m^2^, 2.03 cd/A, and 0.69% at a bias of 6.0 V, respectively. Compared to the performance of CsPbBr_3_ PeLEDs without AAO structures, the peak luminance, current efficiency, and EQE values of CsPbBr_3_ PeLEDs with an AAO structure using a 50 V pore-expanding voltage demonstrated improvements of 282%, 190%, and 1280%, respectively. There are several high performance PeLEDs have published by employed advanced structure to boost the luminance of PeLEDs^27–29^. In this work, the improvement of performance is the scattering owing to the increased surface area of perovskite caused by AAO structure because the shape of AAO structure is rough, according to the SEM images in Figs. 2 and 3. Besides, a larger emission area caused by the random shape of AAO structure also means a larger injection area and to emit more photons as more charges are injected. The EQE of PeLEDs is product of the light output coupling efficiency and internal quantum efficiency (IQE)^30^. Therefore, the improvement of EQE in this work may be attributed to the light output coupling efficiency caused by the scattering of surface and IQE caused by the carrier recombination inside AAO structure. Figure 7 plots the emission diagram of the CsPbBr_3_ active layer without and with the AAO structure using a pore-expanding voltage treatment. The thicknesses of the AAO structures with pore-expanding voltages of 30, 40, and 50 V are approximately 100, 140, and 160 nm, respectively, as shown in Fig. 2d–f. As shown in Fig. 7a, the thickness of the CsPbBr_3_ film formed by spin coating is approximately 95 nm. When using a spectrometer to measure the luminous intensity of an LED, only light in the normal direction (ray A) will be detected by the detector of the spectrometer, and light in the side direction (ray B) will not be detected. The AAO structure allows the light in the side direction to be directed to the normal direction (ray C), which is detected by the detector of the spectrometer. A thicker AAO structure means that more side light is directed to the normal direction, so the luminous intensity is brighter, as shown in Fig. 7b–d. To compare the CsPbBr3 PeLEDs with an EQE over 20%, the current efficiency of this work is lower one order^31,32^. The future work of PeLED should be in capping layer and passivation of non-radiative defects.

## Conclusions

We have demonstrated the performance of CsPbBr_3_ perovskite light-emitting diodes (PeLEDs) with AAO structures. The peak luminance, current efficiency, and EQE were 11,460 cd/m^2^, 2.03 cd/A, and 0.69% at a bias of 6.0 V, respectively. Compared to the performance of CsPbBr_3_ PeLEDs without AAO structures, the peak luminance, current efficiency, and EQE values of CsPbBr_3_ PeLEDs with an AAO structure using a 50 V pore-expanding voltage demonstrated improvements of 282%, 190%, and 1280%, respectively. The performance improvement may be attributed to two factors. One is the scattering owing to the increased surface area of perovskite caused by AAO structure because the shape of AAO structure is rough. The other factor is the increase in effective emission area as a result of the random shape of the AAO structure.

## Data Availability

The datasets used and/or analyzed during the current study available from the corresponding author on reasonable request.
